# Multiple adult xanthogranulomas associated with breast cancer

**DOI:** 10.1016/j.jdcr.2026.04.003

**Published:** 2026-04-08

**Authors:** Samantha Bizimungu, Annie Bélisle, Philippe Watson

**Affiliations:** a Division of Dermatology, Department of Medicine, Centre Hospitalier de l’Université de Montréal, Montréal, Quebec, Canada; b Division of Pathology, Department of Pathology and Cellular Biology, Centre Hospitalier de l'Université de Montréal, Montréal, Quebec, Canada

**Keywords:** breast cancer, cancer-related, dermatoses, histiocytoses, systemic disease, xanthogranuloma

## Introduction

Adult xanthogranuloma (AXG) is a rare non-Langerhans cell histiocytosis of the C group.^1^ It typically presents as a solitary orange hued papule or nodule on the trunk.^2^ A small but growing number of cases of AXG in association with hematologic malignancies have been reported.^3^, ^4^, ^5^ Association with solid organ malignancy is exceedingly rare. In this report we present a case of multiple AXG (MAXG) arising in the setting of infiltrating ductal carcinoma of the breast.

## Case report

**Fig 1:**
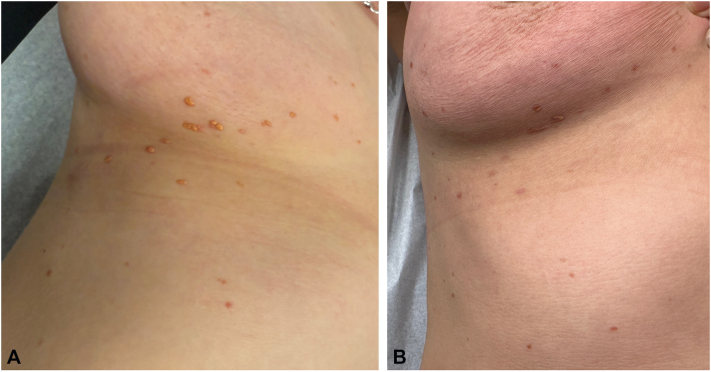
Clinical evolution of xanthogranulomas in a patient with breast cancer. **A,** At baseline, several smooth, orange hued dome-shaped papules are seen on the right breast and trunk. **B,** At 3-month follow-up, flattening and regression of most lesions after initiating neoadjuvant chemotherapy and immunotherapy.

**Fig 2:**
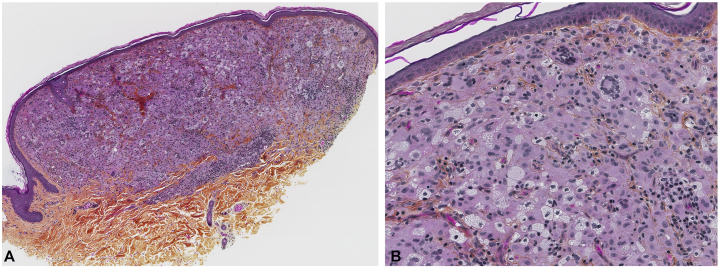
Histopathological image of a xanthogranuloma. **A,** Skin biopsy at scanning magnification (4×) stained with hematoxylin phloxine saffron demonstrating a nodular dermal infiltrate. **B,** Higher magnification (40×) showing xanthomized histiocytes, lymphocytes, and multinucleated giant cells, including some of the Touton type.

A 54-year-old healthy woman presented with a 4-month history of asymptomatic papules on the trunk and upper limbs. Physical examination revealed approximately 40 yellow-orange, smooth, dome-shaped papules clustered on the right breast, where they initially appeared, and scattered on the rest of the trunk and upper limbs (Fig 1, *A*). There was no noticeable involvement of the eyes or oral mucosa. A skin biopsy was performed which demonstrated a dermal nodular infiltrate composed of foamy histiocytes, multiple Touton-type giant cells, and admixed lymphocytes (Fig 2, *A* and *B*). Immunostains showed CD68 positivity and S100 negativity. Immunohistochemistry for BRAF V600E mutation was negative, and next generation sequencing was unavailable. These clinicopathological findings were consistent with a diagnosis of MAXG.

A workup was performed to screen for systemic histiocytic disease and for underlying malignancy. Laboratory evaluation showed normal blood counts, serum protein electrophoresis, lactate dehydrogenase, and liver function tests. A full-body positron emission tomography scan showed no bone lesions but revealed a new hypermetabolic right breast mass and uptake in the right axillary lymph nodes. The patient underwent biopsy of the breast mass which led to a diagnosis of stage III infiltrating ductal carcinoma. She began neoadjuvant treatment for her breast cancer with a standard regimen of carboplatin, paclitaxel and pembrolizumab.

At 3-month follow-up, dermatological examination revealed 50% regression of the skin lesions, leaving behind residual hyperpigmented macules (Fig 1, *B*). Given the described association between AXG and malignancies, as well as the rapid improvement of the lesions with antineoplastic treatment, it is plausible that the patient’s MAXG represent a reactive process to her breast cancer, similar to that described with hematologic cancers.

## Discussion

The xanthogranuloma family is a group of non-Langerhans cell histiocytoses belonging to the C group.^1^ Juvenile xanthogranuloma is the most common form, presenting as a single or multiple red to yellow nodules which resolve spontaneously within months to years. AXG is rare and typically presents as a persistent, solitary lesion on the trunk.^2^ MAXG is defined by the presence of 5 or more lesions, and may present with systemic involvement.^6^ Somatic mutations in the mitogen-activated protein kinase pathway have been identified in some cases, and it has been suggested that XG-related disorders exist on a spectrum with Erdheim-Chester disease.^7^ MAXG has also been associated with underlying malignancy.^2^ To date, 11 cases of AXG in association with hematologic malignancies have been reported in the literature.^4^ The only previously published case of a solid tumor in association with AXG is a report of MAXG in a patient with gastrointestinal stromal tumor.^8^

We report a unique case of MAXG in association with infiltrating ductal carcinoma of the breast. The temporal relationship between the onset of the cutaneous lesions and diagnosis of the breast tumor, clustering of lesions around the affected breast, and regression of lesions with treatment of the cancer are in favor of a reactive process secondary to the neoplasm. Clinicians should be alerted to the possibility of an underlying solid tumor or hematologic malignancy in the setting of MAXG. As such, it is important to thoroughly evaluate patients with MAXG for the presence of an underlying neoplastic process.

## Conflicts of interest

None disclosed.
